# Supplementary material for: Characterization of subclonal variants in HG002 Genome in a Bottle reference material as a resource for benchmarking variant callers
Source: Cell Genom. 2025 Dec 19;6(4):101104. doi: 10.1016/j.xgen.2025.101104 (PMC13069850; doi:10.1016/j.xgen.2025.101104)
Supplement: Document S1. Figures S1–S5 and Tables S1, S3, and S4 [file mmc1.pdf]

Cell Genomics, Volume *6*

Supplemental information

Characterization of subclonal variants

in HG002 Genome in a Bottle reference material

as a resource for benchmarking variant callers

Camille A. Daniels, Adetola A. Abdulkadir, Megan H. Cleveland, Jennifer H. McDaniel, David Jáspez, Luis Alberto Rubio-Rodríguez, Adrián Muñoz-Barrera, José Miguel Lorenzo-Salazar, Carlos Flores, Byunggil Yoo, Sayed Mohammad Ebrahim Sahraeian, Yina Wang, Massimiliano Rossi, Arun Visvanath, Lisa Murray, Wei-Ting Chen, Severine Catreux, James Han, Rami Mehio, Gavin Parnaby, Andrew Carroll, Pi-Chuan Chang, Kishwar Shafin, Daniel Cook, Alexey Kolesnikov, Lucas Brambrink, Mohammed Faizal Eeman Mootor, Yash Patel, Takafumi N. Yamaguchi, Paul C. Boutros, Karolina Sienkiewicz, Jonathan Foox, Christopher E. Mason, Bryan R. Lajoie, Carlos A. Ruiz-Perez, Semyon Kruglyak, Justin M. Zook, and Nathan D. Olson

Characterization of subclonal variants in HG002 Genome In A Bottle reference material as a resource for benchmarking variant callers.

Supplemental Figures and Tables

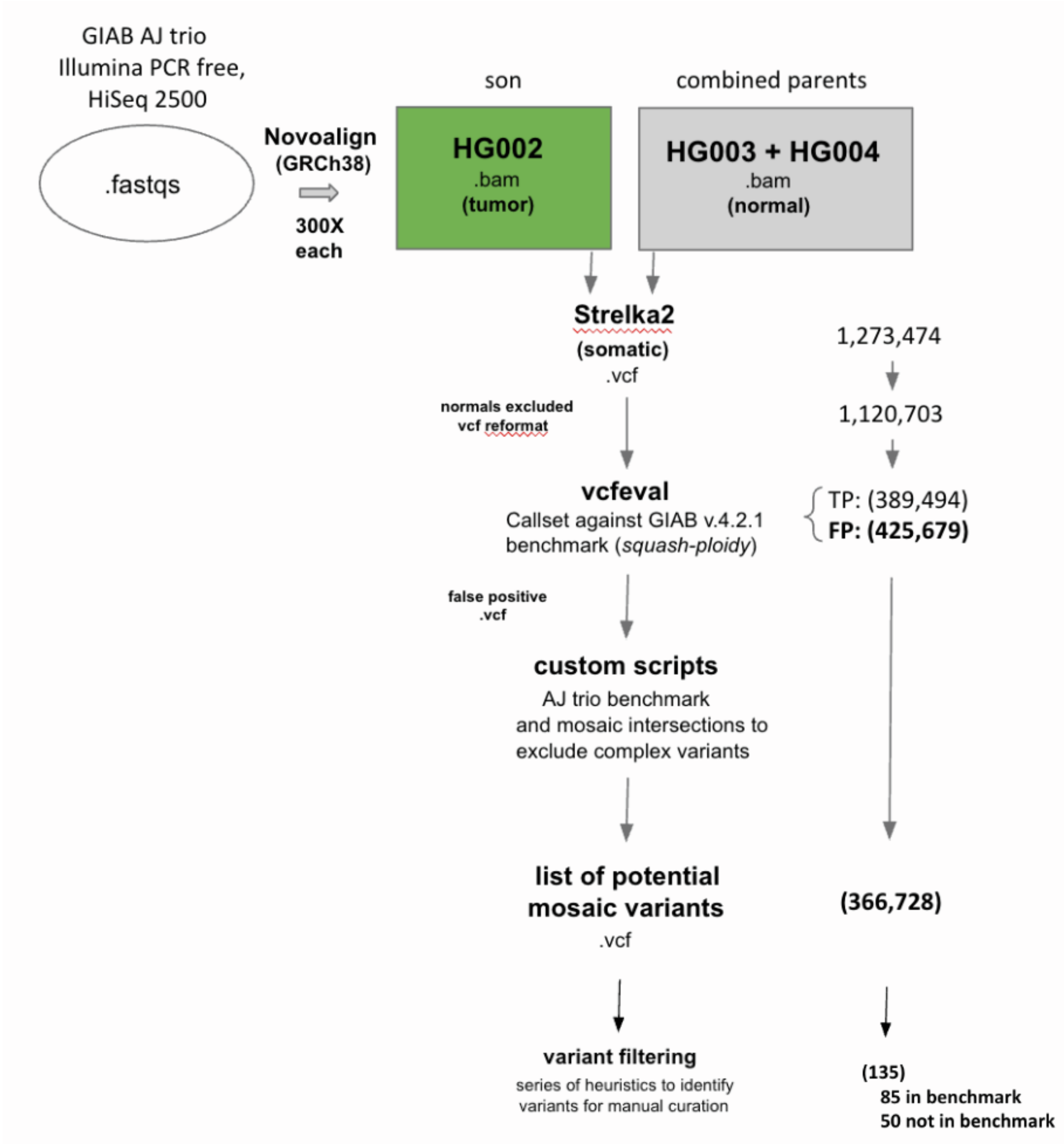

Figure S1: Steps for filtering potential mosaic variants, related to Star Methods. Variant counts from the Strelka2 tumor-normal run with the GIAB AJ trio, benchmarking, and filtering steps to generate a list of potential mosaic variants (366,728 vcfeval false positives) for database creation.

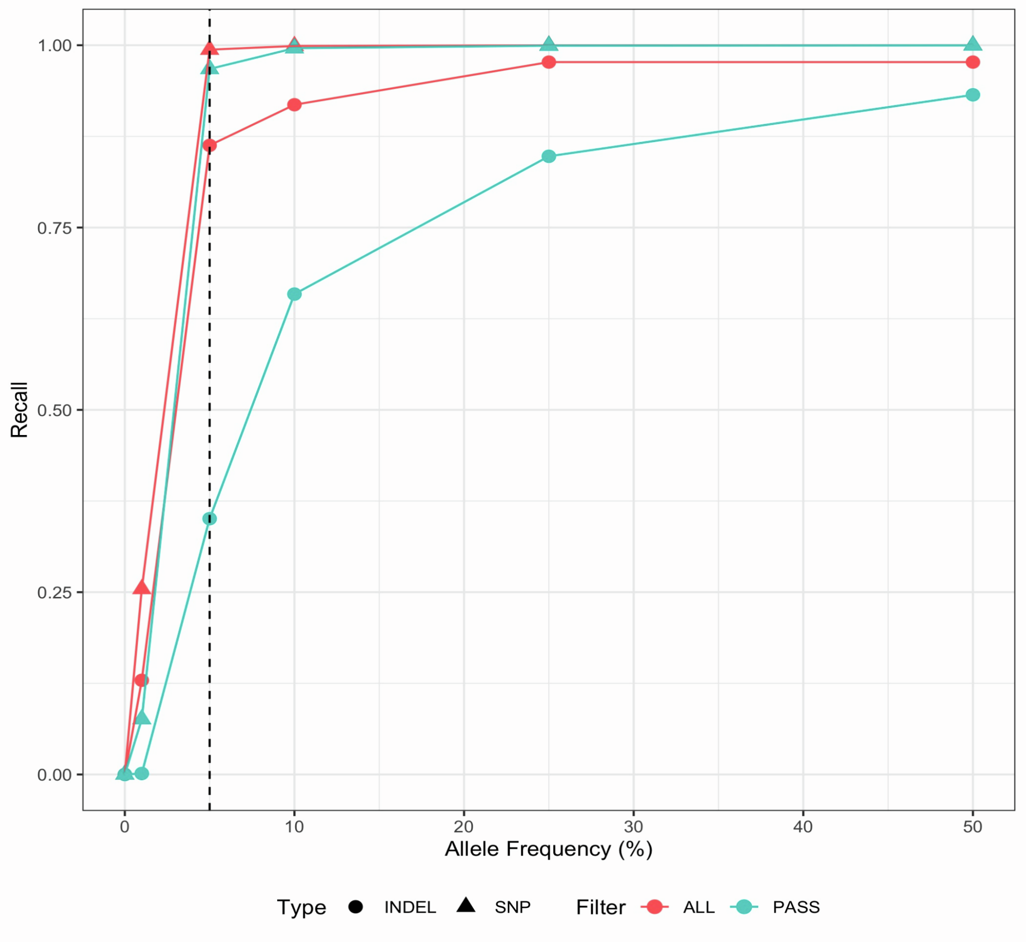

Figure S2: Limit of detection (LOD) determination, related to Figure 1. Limit of detection (LOD) was established at 5% variant allele fraction (VAF) using Strelka2 callsets from six in-silico mixtures of GIAB reference 300x samples (subset to chromosome 20), HG002 (son) and HG003 (father), as a control. X-axis values indicate the different samples, ranging from 0% AF (HG003 unmixed) to 50% AF (HG002 unmixed). Callsets for each mixture were benchmarked in the precisionFDA app.

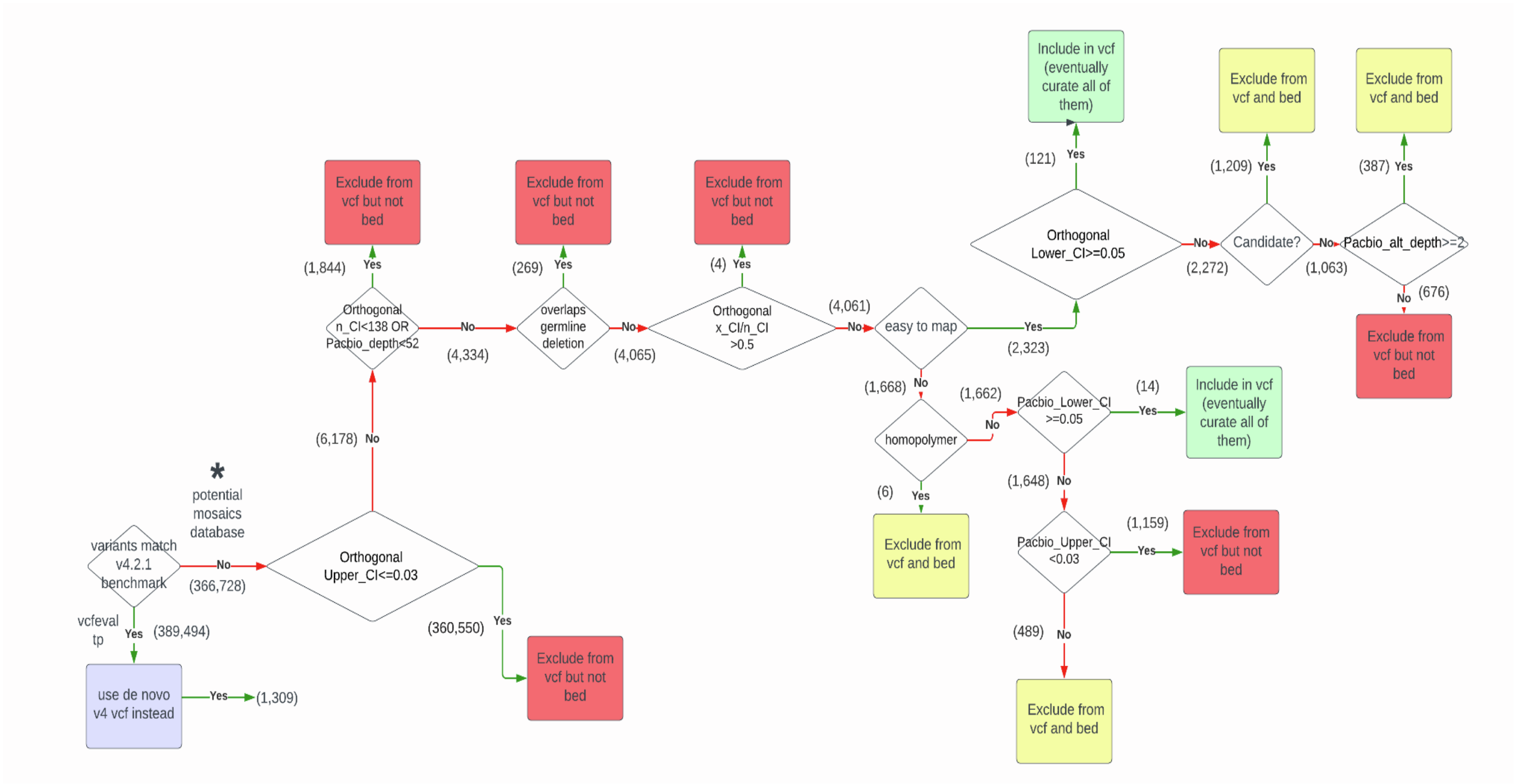

Figure S3: Mosaic benchmark decision tree for filtering potential mosaic variants to produce a list for manual curation, related to Star Methods. Mosaic benchmark decision tree for filtering potential mosaic variants to produce a list for manual curation. A series of heuristics were applied to the potential mosaic database (* starting at bottom left) using combined orthogonal CI thresholds and other attributes for candidate set determination. The tree resulted in three groups of variants: green boxes indicate variants to curate, yellow represents variants excluded from benchmark VCF and BED, and red variants excluded from benchmark VCF but not BED. Values in parentheses are variant counts for each step of the decision tree.

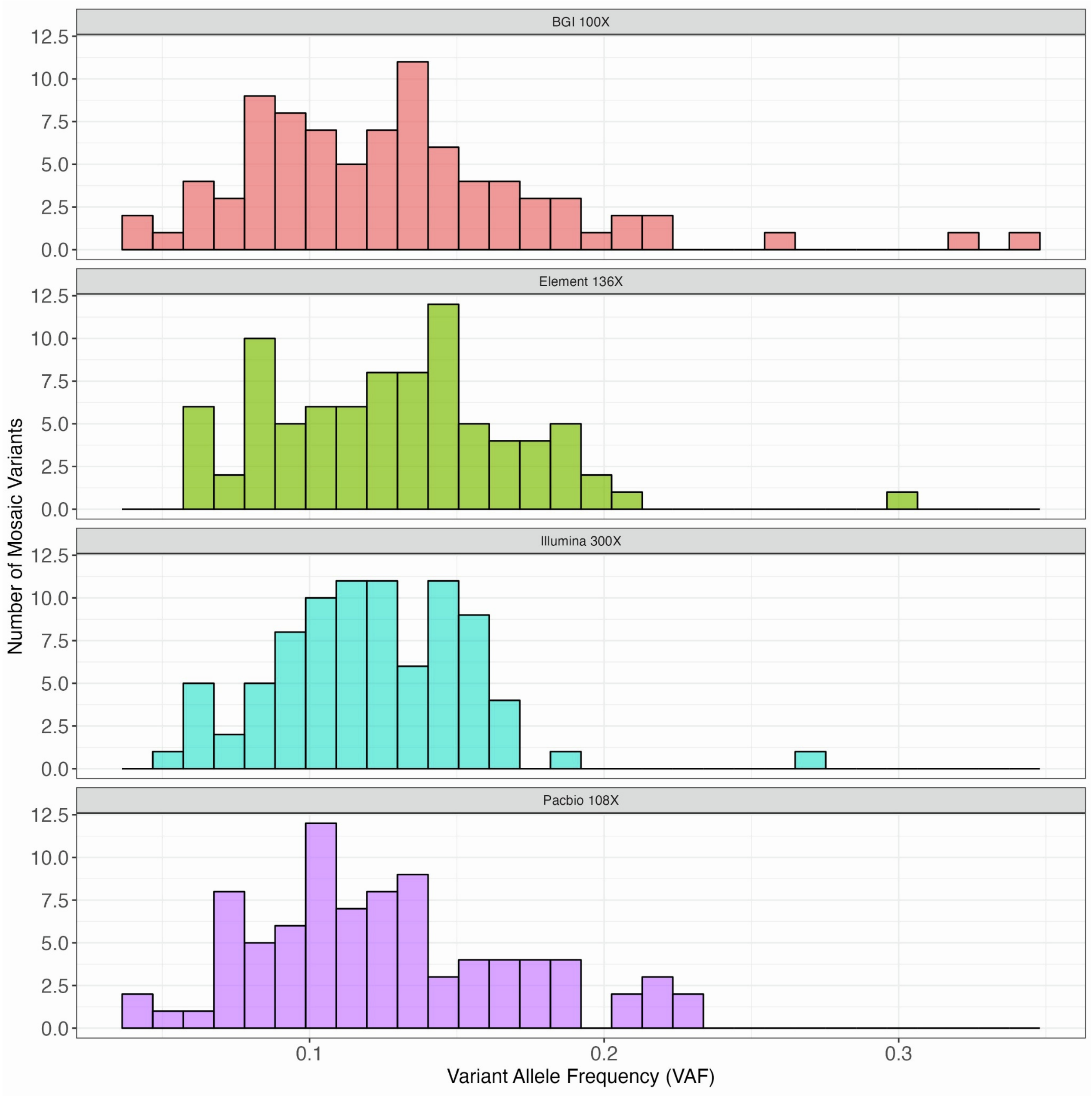

Figure S4: Mosaic variant VAFs, related to Figure 2. distributions for the 85 HG002 **mosaic benchmark variants** in high-coverage Illumina (300x) and orthogonal (BGI - 100x, Element - 136x, PacBio HiFi - 106x) datasets generated from NIST HG002 reference material.

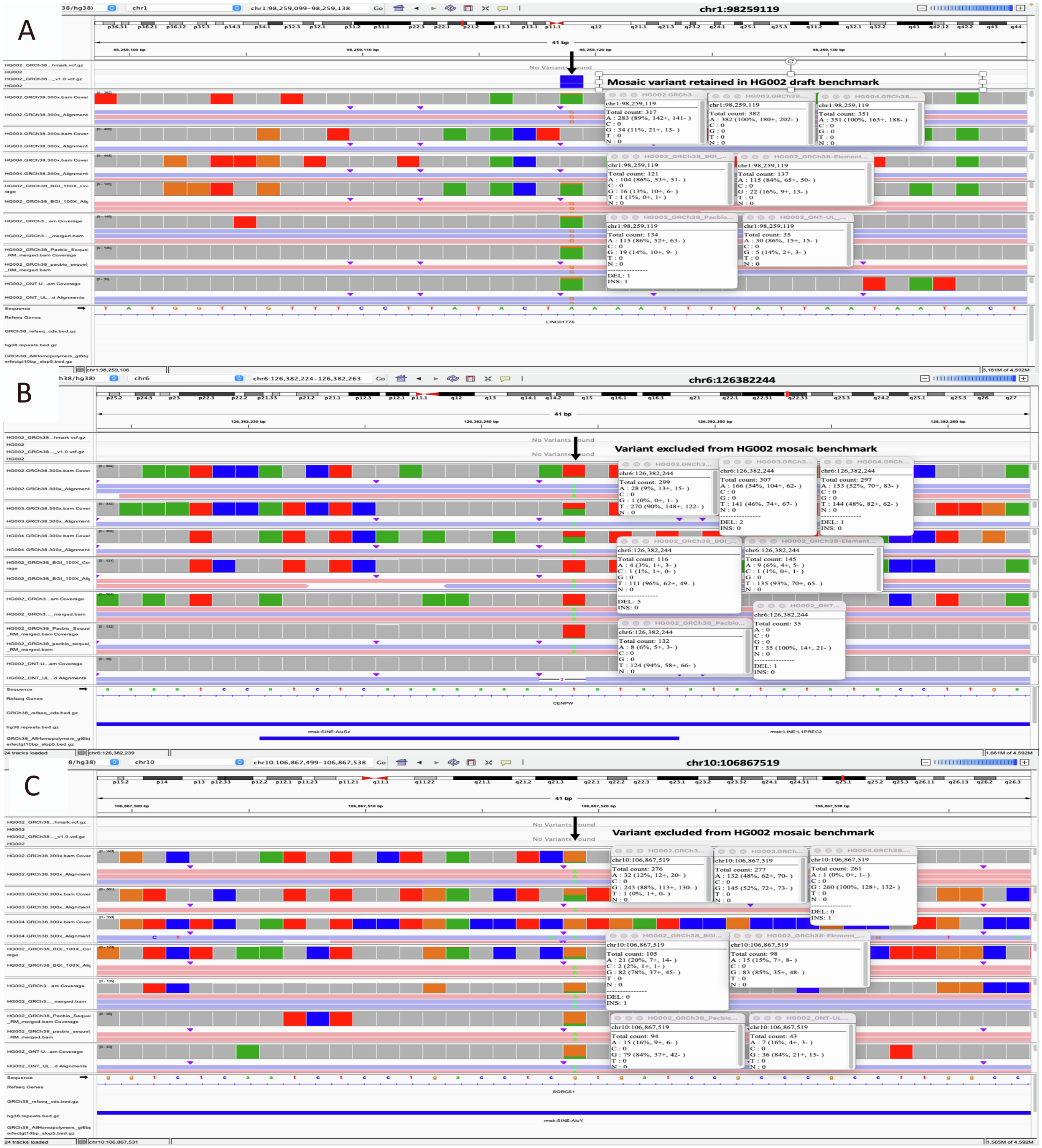

Figure S5: IGV profiles of variants (A) included in and (B, C) excluded from the HG002 mosaic benchmark after manual curation, related to Star Methods. IGV profiles of variants (A) included in and (B,C) excluded from the HG002 mosaic benchmark after manual curation. Profile A depicts an HG002-specific candidate mosaic SNV (chr1:98259119 - not detected in parents), while B and C show a putative and candidate SNV (chr6:126382244 and chr10:106867519). The first three data tracks are the HG002 (son), HG003 (mother), and HG004 (father) 300X coverage Illumina data. The following tracks are for HG002 BGIseq dataset, Element, PacBio Sequel, and ONT-UL datasets.

Table S1: Variant allele fraction counts for the potential HG002 mosaic variant database, related to Figure 1. Variant allele fraction counts for the potential HG002 mosaic variant database. Bold text indicates VAF bins targeted for inclusion in the benchmark set. Candidate variants passed the Strelka2 filter and putative variants were filtered.

VAF Bin	*Bin Count*	*SNV Candidate*	*SNV Putative*	*Indel Candidate*	*Indel Putative*
0%	230,020	0	230,020	0	0
0-1%	97,944	0	97,944	0	0
1-4%	19,177	1,460	17,712	5	0
4-5%	1,828	158	1,668	2	0
5-10%	7,153	100	7,052	1	0
10-30%	8,768	72	8,691	5	0
>30%	1,738	126	1,604	2	0
Total	366,728	1,916	364,791	15	6

Table S3: Medically relevant genes in the HG002 mosaic benchmark variants, related to Star Methods. HG002 mosaic benchmark variants (13) that overlap medically relevant genes (MRGs) and number of bases in mosaic benchmark regions and fraction of genes covered by benchmark regions.

**CHR**	**POS**	**REF**	**ALT**	**GENE**	**# Bases Covered**	**# Bases Covered**
chr2	178057258	A	G	*PDE11A*	413,145	0.919
chr2	215348334	A	G	*ATIC*	36,632	0.968
chr5	151835516	A	G	*GLRA1*	99,031	0.967
chr7	110674520	A	G	*IMMP2L*	875,644	0.973
chr8	69549454	G	T	*SULF1*	188,531	0.97
chr8	112611526	T	A	*CSMD3*	1,178,753	0.97
chr9	95143456	C	A	*FANCC*	318,626	0.972
chr10	18475692	A	T	*CACNB2*	380,600	0.944
chr10	27062623	T	A	*ANKRD26*	101,653	0.936
chr10	67598410	C	A	*CTNNA3*	1,723,122	0.966
chr14	55605762	A	C	*KTN1*	131,637	0.924
chr15	47607398	C	T	*SEMA6D*	575,813	0.975
chr21	40437618	A	C	*DSCAM*	765,587	0.915

Table S4: External somatic callset comparisons against the HG002 mosaic benchmark v1.0, related to Table 1. External somatic callsets and hap.py results from comparisons against the HG002 mosaic benchmark v1.0. The benchmark set contains 85 high confidence mosaic SNVs. SUPPORTED column represents the number of true positives (TP) from a mosaic v1.0 benchmark comparison and EXTRA columns represent query - false positives (FP) from a combined mosaic v1.0 + GIAB v4.2.1 small variant benchmark comparison are reported for passing SNVs. Some FPs may be true mosaic variants at low VAF. >=Q0 and >=Q10 represent variant quality score thresholds for a specified callset. Note that these comparisons were intended to evaluate the accuracy of the benchmark and not evaluate performance of each method, because the participants were not blinded, some methods were experimental, and most methods are under active development. +In addition, PacBio Revio sequencing was not from the NIST RM DNA, so many putative FPs appear to be true mosaic variants >5% VAF in their cell line and not in the NIST RM DNA.

Group	*Data*	*Cov.*	*Caller*	*Mode*	*Supported*	*PASS*	*5-30%VAF*	*Selection*	*# Curated*
Byunggil Yoo	Illumina	40x	DRAGENv4.0.3	TO	21	112765<br>9	1914*	RAND	10
Cornell	Illumina	300x	Sentieon v.202010	TO	74	21	13	EXTRA	13
	Illumina	300x	Sentieon v.20230801	T/N	79	47	20*	EXTRA	20
Element	Element	70x	DeepSomatic v1.6.1	T/N	81	318	273*	QUAL	4
DRAGEN	Illumina	300x	DRAGENv4.3.6	TO g+m	85	428	105*	QUAL	9
	Illumina	300x	DRAGENv4.3.6 mosaics only	TO g+m	85	187	41*	QUAL	6
Google	Illumina	300x	DeepSomatic, q0	T/N	84	3504	2530*	QUAL	28
	Illumina	300x	DeepSomatic, q10	T/N	79	970	720*	QUAL	-
	Element	100x	DeepSomatic, q0	T/N	81	2817	1950*	QUAL	6
	Element	100x	DeepSomatic, q10	T/N	78	511	436*	QUAL	-
	Onso	35x	DeepSomatic, q0	T/N	45	297	205**	QUAL	8
	Revio+	130x	DeepSomatic, q0	T/N	83	1557	1232	QUAL	17
	Revio+	130x	DeepSomatic, q10	T/N	81	935	885	QUAL	-
ITER	Illumina	300x	DRAGENv4.2.4	TO	69	888	135	RAND	10
	Illumina	300x	Mutect2 (GATK4)	TO	82	248397	80965	RAND	10
	Illumina	300x	Strelka 2.9.10 (HG4 Norm)	T/N	81	892	399*	RAND	10
	Illumina	300x	Strelka 2.9.10 (HG4 Norm)	T/N	81	892	442*	RAND	10
Roche	Illumina	300x	NeuSomatic v0.1.4	T/N	83	1	1*	EXTRA	1
UCLA	Illumina	300x	Consensus	T/N	84	27	1*	EXTRA	1
